# Potential Role of Tumor-Derived Exosomes in Non-Small-Cell Lung Cancer in the Era of Immunotherapy

**DOI:** 10.3390/life12122104

**Published:** 2022-12-14

**Authors:** Alfredo Tartarone, Rosa Lerose, Marina Tartarone, Michele Aieta

**Affiliations:** 1 Department of Onco-Hematology, Division of Medical Oncology, 85028 Rionero in Vulture, Italy; 2 Hospital Pharmacy, IRCCS-CROB Referral Cancer Center of Basilicata, 85028 Rionero in Vulture, Italy; 3 Faculty of Medicine, Humanitas University, 20090 Rozzano, Italy

**Keywords:** non-small-cell lung cancer, exosomes, tumor-derived exosomes, immune-checkpoint inhibitors, liquid biopsy

## Abstract

Lung cancer, of which non-small-cell lung cancer (NSCLC) represents about 80% of all cases, is the second most common cancer diagnosed in the general population and one of the major causes of cancer-related deaths worldwide. Overall, the outcomes of patients with advanced NSCLC are still disappointing despite advances in diagnosis and treatment. In recent years immune-checkpoint inhibitors (ICIs), administered alone or in combination with chemotherapy, have revolutionized the treatment landscape of patients with advanced non-small-cell lung cancer. However, until now, tissue expression of PD-L1 and tumor mutation burden represent the only available biomarkers for NSCLC patients treated with ICIs. A growing body of evidence showed that tumor-derived exosomes (TDEs) have the PD-L1 protein on their surface and that they are involved in angiogenesis, tumor growth, invasion, metastasis and immune escape. This review focused on the potential clinical applications of TDEs in NSCLC, including their possible role as a biomarker for prognosis and disease monitoring in patients undergoing immunotherapy.

## 1. Introduction

Lung cancer, of which non-small-cell lung cancer (NSCLC) represents about 80% of all cases, is the second most common cancer diagnosed in the general population and the first leading cause of cancer-related death in the United States, [1]. In addition, NSCLC is normally diagnosed at a late stage (A-NSCLC). In the United States lung cancer incidence declined from 2009 to 2018 by 1% annually in women and 3% in men, reflecting temporal trends in smoking prevalence [1]. The three most common types of NSCLC are adenocarcinoma, which represents about 50% of all NSCLC diagnoses, squamous cell carcinoma and large cell carcinoma, accounting for 30% and 10% of all diagnoses, respectively. Squamous cell carcinoma is more closely associated with smoking than adenocarcinoma and large cell carcinoma.

At present, the outcomes of patients with A-NSCLC are still disappointing despite the availability of both newer biological knowledge and various novel therapies, considering that the current five year relative survival rate for all stages combined is 22% [1]. In this scenario, the most important advances in recent years concerning the treatment of A-NSCLC are represented by the arrival of targeted therapy (e.g., osimertinib, alectinib, entrectinib, selpercatinib, sotorasib and dabrafenib/trametinib) in patients with oncogene-addicted disease and by the advent of immunotherapy, administered alone or in combination with chemotherapy, in patients with non-oncogeneaddicted disease. Common types of immunotherapy agents include the immune checkpoint inhibitors (ICIs), such as monoclonal antibodies (mAbs) anti PD-1 and anti PD-L1 that can interrupt inhibitory immune signals and restore the antitumor immune response. Immune checkpoints are part of our immune system and have a role in preventing the destruction of healthy cells caused by a strong immune response. Programmed cell death protein 1 (PD-1) is an immune checkpoint protein localized on the surface of many peripheral activated cells of the immune system, such as natural killer T cells, CD4+, CD8+ T-cells and B cells [2]. The PD-1 receptor possesses two ligands, the programmed cell death ligand 1 (PD-L1) and the programmed cell death ligand 2 (PD-L2), released by neoplastic and stromal cells; generally, PD-L1 expression is induced by the intrinsic and extrinsic signals of tumor cells. The PD-1 pathway has a crucial role in preventing autoimmunity and in the maintenance of self-tolerance; however, tumor cells are able to escape the immune system by exploiting these immune checkpoints. To date, several mAbs targeting PD-L1/PD-1 were approved for the treatment of A-NSCLC, including nivolumab, pembrolizumab, atezolizumab, durvalumab and cemiplimab [3].

In recent years the above mentioned ICIs, administered as a monotherapy or in combination with a chemotherapy, have revolutionized the treatment landscape of A-NSCLC by significantly prolonging the overall survival (OS) in first- and second-line settings [4,5,6,7]. Until now, immunohistochemistry (IHC) PD-L1 expression and tumor mutation burden (TMB) represent the only available biomarkers for NSCLC patients treated with ICIs. However considering that, mainly due to tumor immune-escape mechanisms exploited by cancer and/or tumor heterogeneity, some patients with high PD-L1 expression do not benefit from ICIs as well as some patients with a low or negative PD-L1 expression; hence, there is an urgent need to identify other sources of both biomarkers and novel biomarkers with the aim to improve the selection of patients eligible for immunotherapy.

Recently, in the context of a liquid biopsy, exosomes have received special attention for their role in facilitating early diagnosis and improving the treatment outcomes in cancer patients. Moreover, as we will see later, several studies indicate that proteins, lipids and nucleic acids, carried by tumor-derived exosomes (TDEs), have the same characteristics of parental cells and can represent a novel and promising source of biomarkers. Regarding PD-L1, it was proven that in cancer patients it can be found in extracellular forms, such as exosomes or as a freely soluble protein, and it has also been reported that exosomal PD-L1 (exoPD-L1) is generally more stable than the soluble form and more simply available than the membranous type [8].

In addition, growing evidence indicates that freely circulating and extracellular vesicles (EVs)—microRNAs (miRNAs), can play a role as predictive or prognostic biomarkers in response to antitumoral treatments in several types of solid cancers, including NSCLC, even though EVs represent a better source of miRNAs for biomarker studies in terms of stability, quantity and quality when compared to freely circulating miRNA [9]. Around the world, researchers are working to discover biomarkers that can identify which cancer patients are likely to respond to immunotherapy. For example, in the last few years in the United States of America (USA) the National Cancer Institute (NCI) has created a network of four research institutions (Dana-Farber Cancer Institute, Icahn School of Medicine at Mount Sinai, MD Anderson Cancer Center and Stanford University) named Cancer Immune Monitoring and Analysis Centers (CIMACs) with the aim to develop new biomarkers for immunotherapy, and a storage center–the Cancer Immunologic Data Commons (CIDC), which is also at Dana-Farber Cancer Institute.

In this paper we reviewed the main clinical experiences reported in the literature regarding, in particular, the possible role of TDEs as potential sources of novel biomarkers in cancer patients undergoing immunotherapy.

## 2. Exosomes and Tumor-Derived Exosomes

Exosomes were described for the first time in the 1980s when some researchers described their release into the extracellular space of transferrin receptors from the membrane of maturing erythroblasts and reticulocytes as small vesicles [10,11,12].

Since their discovery 40 years ago, in the scientific world it became increasingly clear that exosomes have a key role in many aspects of physiology and disease, including the intercellular signaling crosstalk and tumor growth. Exosomes are small EVs detectable in all body fluids (blood, urine, saliva, tears, etc.) released by many kinds of cells (cancer cells, reticulocytes, dendritic cells, lymphocytes, etc.) both in physiological and pathological conditions.

**Figure 1:**
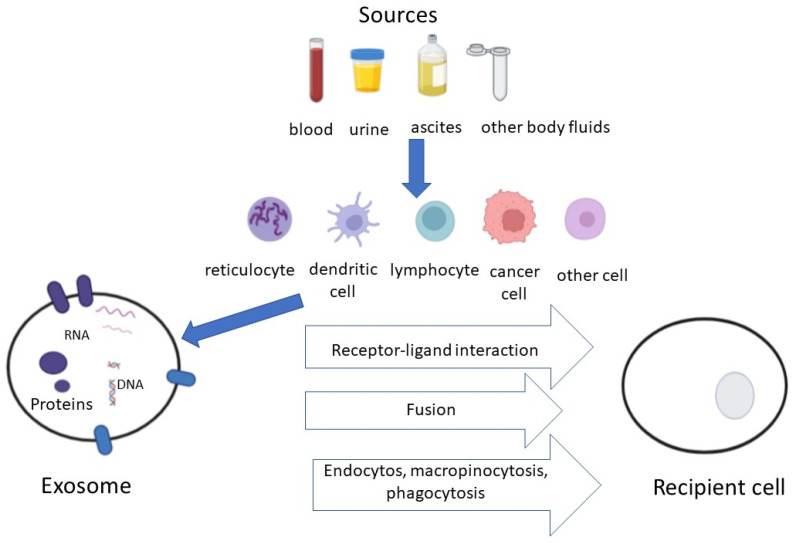
(Modified from Zheng et al. [15]). Exosomes are nano-sized (30–150 nm) extracellular vesicles secreted via exocytosis from different cell types, including cancer cells. They are taken up by target cells, which communicate information via surface protein signaling as well as through the transfer of nucleic acids, lipids and other molecules.

In more detail, the term EVs should be used to describe non-replicating structures naturally released by the parental cells that are delimited by a lipid bilayer as reported in the current minimal information for studies of extracellular vesicles (MISEV) guidelines [13]. Exosomes are EVs of endosomal origin that range in size between 30 and 150 nm. They carry various molecules that derive from parent cells, such as proteins (enzymes, receptors and extracellular matrix proteins), nucleic acids (mRNA, miRNA and DNA) and lipids (Figure 1) [14,15]. EVs with their content of lipids, proteins and nucleic acids can affect the physiology of the recipient cells [16]. The mechanism of EVs release derives from a complex signaling network that includes Rab-GTPase family members and soluble N-ethylmaleimide sensitive factor (NSF) and attachment protein receptors (SNAREs) [17]. The biogenesis of exosomes is a dynamic multistep process characterized by the maturation of early endosomes into late endosomes, formation of intraluminal vesicles (ILVs) within multivesicular bodies large (MVBs) and the extracellular release of exosomes [18]. It is recognized that tumor cells liberate more exosomes than normal cells and that the surface of TDEs contains membrane proteins, such as EGFR, CD317, CD91 as well as PD-L1, which might represent potential tumor markers [19,20].

Moreover, a growing body of evidence suggests that TDEs are mediators of intercellular communication and have a role in angiogenesis, tumor growth, invasion and metastasis and in immune escape [21,22,23] (Table 1). TDEs, in short, represent a “mini biopsy” of the tumor and, thanks to the lipid bilayer that protects their molecular content from degradation by nucleases and proteases, they can transfer a series of information into the recipient cells. TDEs represent the cell of origin in many aspects. For example, Thakur et al. assessed EGFR mutations in exosomal DNA (exoDNA) from several NSCLC cell lines, including H292 (EGFR WT), H1975 (harboring the L858R and T790M mutations), H1650 and PC-9 (harboring the exon 19 deletion) [24]. They demonstrated that the exoDNA deriving from lung tumor cells in culture reflected the mutational status of the parenteral cell lines. The same authors utilized a pre-clinical animal model of melanoma (SK-MEL-28) expressing the BRAF (V600E) mutation and found the V600E mutation in the circulating exoDNA isolated from melanoma-bearing mice. 

ExoPD-L1 from tumors is capable in suppressing antitumor immunity through ligation of PD-1 on T cells both locally and systemically by facilitating immune escape and tumor progression [25]. For example, Kim et al. demonstrated that exosomes from lung cancer cells express PD-L1 and contribute to immune escape by decreasing T-cell activity and accelerating tumor growth [25]. Other researchers showed that the growth of PD-L1-positive tumors at distant foci is inhibited when exoPD-L1-deleted tumor cells are coinjected into mice simultaneously or months later [26]. Conceivably, the most promising therapeutic imputation of these findings is that by inhibiting exosome secretion at one tumor site, a systemic and durable immune response against secondary tumors or distant tumor sites arises, similarly to the abscopal effect sometimes observed in patients treated with radiotherapy.

Chinese researchers investigated the role of plasma exosomal total protein (exo-pro) and plasma exosomal T-cell immunoglobulin and mucin-domain-containing molecule 3 (Tim-3) and Galectin-9 (exo-T/G) in 103 NSCLC patients and in 56 healthy subjects [27]. They showed that exo-pro, exo-T and exo-G were significantly increased in NSCLC plasma with respect to healthy samples; high levels of exo-T and exo-G were positively correlated with multiple malignant criteria, including larger tumor dimension, lymph node metastasis and distant metastases; additionally, exo-T and exo-G were higher in plasma samples from lung squamous cell carcinoma when compared to those from lung adenocarcinoma. Fan et al. retroactively evaluated the prognostic value of exoPD-L1 and sPD-L1 in preoperative plasma of 69 gastric cancer (GC) patients and in 31 metastatic GC patients before chemotherapy [28]. The OS was remarkably lower in the high exoPD-L1 group when compared to the low exoPD-L1 group, while sPD-L1 showed no correlation with the OS. In addition, exoPD-L1 presence in the plasma samples of 31 metastatic GC patients was negatively correlated with the CD4+ T cell count, CD8+ T-cell count and granzyme B, suggesting that exoPD-L1 was linked to the immunosuppressive status of GC patients. 

Recently, great interest has been focused on exosomes containing exo-miRNAs for their capacity to regulate post-transcriptionally gene expression. It has also been shown that tumor-derived miRNAs mediate changes in the tumor microenvironment, are crucial in the cross talk between cancer cells and immune cells and may be involved in tumor development [29]. Additionally, exo-miRNAs could represent a potential biomarker for lung cancer because of their stability and specificity. In fact, miRNAs enclosed in exosomes are physically secured from RNAse degradation and their blood concentration is usually high. 

Huang et al. found that the expression of miR-34c-3p was remarkably downregulated in exosomes from NSCLC patients in comparison with that of normal controls; they also demonstrated that exosomes carrying low levels of miR-34c-3p can speed up the migration and invasion of NSCLC by upregulating integrin α2β1 [30]. Liu et al. discovered that high levels of exosomal miR-23b-3p, miR-10b-5p and miR-21-5p were independently correlated with a poor OS in NSCLC patients and could be considered as promising prognostic biomarkers [31]. Cazzoli et al. considered the expression levels of plasma exosomal miRNAs from 10 patients affected by lung adenocarcinoma, 10 patients with pulmonary granuloma and 10 healthy smokers [32]. Afterwards, selected microRNAs were evaluated on a larger group of samples. They demonstrated that exosomal microRNAs (miR-378a, miR-379 etc.) can be employed to distinguish lung cancer patients from healthy people and from pulmonary granuloma patients (miR-151a-5p, mir-154-3p etc.). More recently, other Italian researchers evaluated the combined role of exo-miRNAs and peptidome, in combination with clinical parameters, to prognosticate disease recurrence of a series of early-stage NSCLC patients that had been surgically treated [33]. They found out that the combination of exo-miR-130a-3p with fibrinopeptide A is strongly associated with disease free survival (DFS) and can identify a group of high-risk patients. The authors concluded that these outcomes support the proof-of-concept for integrating circulating markers to ameliorate a patient’s risk score. The drug resistance information passed on by exo-miRNAs represents another interesting area of study. Li et al. demonstrated that exosome-derived miR-184 and miR-3913-5p expression levels increased prominently after the onset of osimertinib resistance and suggested that they could represent novel biomarkers to indicate osimertinib resistance [34]. Finally, considering that TDEs influence many processes associated with tumor growth, they could play a role also in anti-cancer treatment. More precisely, exosomes are being studied as potential cancer vaccines or as carriers of antineoplastic agents (e.g., chemotherapeutics and monoclonal antibodies); additionally, exosomes produced by macrophages may be used to treat cancer [35]. 

Table 2 summarizes the potential theranostic (therapeutic and diagnostic) applications of exosomes in NSCLC.

## 3. Methods for the Isolation of Exosomes

As listed in Table 3, several methods for the isolation of exosomes from biological fluids are available, including ultracentrifugation, ultrafiltration, chromatography, polymer precipitation and immumomagnetic beads [38]. Protein markers expressed on the surface of exosomes, such as CD9, CD63 and CD81 can be used for the identification of exosomes. In any case, the International Society of Extracellular Vesicles (ISEV) published a series of recommendations for the study of EVs [13].

Methods for the detection of exoPD-L1 in clinical samples include Enzyme Linked Immunosorbent Assay (ELISA), that at present is the most used method, Homogeneous Low-Volume Efficient and Sensitive (HOLMES)-exoPD-L1, Nano Plasmonic Exosome assay (nPLEX), Surface Enhanced Raman Scattering (SERS) and flow cytometry. Along with the improvement of novel technologies other methods are in development; however, it should be noted that the validation of these methods in large cohorts represents a necessary condition before routinely using exoPD-L1 as a clinical biomarker.

MicroRNAs can be extracted from biological fluids via the enzyme linked immunosorbent assay (ELISA) or a Western blot analysis with next-generation sequencing (NGS) and real time polymerase chain reaction (RT-PCR) [19].

## 4. Tumor-Derived Exosomes/Micro-RNAS and Immunotherapy: Data from the Literature

Until now, few studies have assessed the possible role of PD-L1 expression in TDEs as a predicting biomarker in cancer patients undergoing ICIs. Yang et al. conducted a study to evaluate the correlation between tumor tissue PD-L1 (tPD-L1) and blood PD-L1 (bPD-L1), including PD-L1 mRNA, exoPD-L1 and soluble PD-L1(sPD-L1) and to monitor their changes during ICIs treatment in 51 cancer patients of which 40 had advanced NSCLC [39]. The authors showed a positive association between tPD-L1 and bPD-L1; moreover, patients with both a fold change in PD-L1 mRNA ≥ 2.04 and a fold change in exoPD-L1 ≥ 1.86 reported the best efficacy and OS results. Li et al. evaluated the clinical significance of PD-L1 expression in serum derived exosomes in 85 NSCLC patients [40]. They reported that exoPD-L1, but not sPD-L1, was associated with NSCLC disease progression as well as lymph node status, tumor dimension, metastasis and stage; however, exoPD-L1 was not linked to PD-L1 IHC status. Del Re et al. showed both the feasibility of PD-L1 mRNA measurement in plasma-derived exosomes and its association with response to ICIs in patients with melanoma and NSCLC [41]. In particular, they demonstrated a significant decrease in PD-L1 mRNA levels in plasma derived exosomes of patients responding to therapy and an increase in it in patients with disease progression. Okuma et al. prospectively evaluated the baseline plasma sPD-L1 levels from 39 NSCLC patients using an enzyme-linked immunosorbent assay [42]. They found that 59% of patients with low plasma sPD-L1 levels and 25% of those with elevated plasma sPD-L1 levels achieved a partial or a complete response, respectively; additionally, 75% of patients with high plasma sPD-L1 levels developed progressive disease when compared with 22% of those with low plasma sPD-L1 levels. The authors concluded that plasma sPD-L1 levels may represent a novel biomarker for the prediction of the efficacy of nivolumab therapy in NSCLC. Recently, de Miguel Perez et al. evaluated the prognostic role of plasma EV PD-L1 expression in a retrospective cohort of 33 NSCLC patients treated with ICIs and validated it in a prospective analysis of a cohort of 39 patients enrolled in the phase 2 PROLUNG trial with pembrolizumab and docetaxel or docetaxel alone [43]. More precisely, they analyzed the EV PD-L1 levels from baseline to eight weeks of ICI therapy and observed that non-responders had increased levels in comparison to a decrease in responders. Peng et al. evaluated the possibility of utilizing plasma exosomal microRNAs as a biomarker in 30 NSCLC patients that received immunotherapy [44]. Plasma samples of these patients were gathered before the administration of ICIs and after every three cycles until disease progression. In this study, three miRNAs from hsa-miR-320 family (hsa-miR-320d, hsa-miR-320c, and hsa-miR-320b) were identified as potential predictors of response considering that they remarkably upregulated in the PD groups when compared with the PR group at baseline before the therapy. Furthermore, has-miR-125b-5p was found to be downregulated in patients responding to ICIs; therefore, the authors suggested that a continuous drop in the level of has-miR-125b-5p during the ICIs treatment could be a useful predictor for better outcomes. Utilizing next generation sequencing (NGS), Halvorsen et al. performed a miRNA profiling in serum samples collected from NSCLC patients (*n* = 20) prior to initiation of immune therapy with nivolumab [45]. They identified a signature of seven microRNAs (miR-215-5p, miR-411-3p, miR-493-5p, miR-494-3p, miR-495-3p, miR-548j-5p and miR-93-3p) significantly associated with an OS > 6 months. Boeri et al. prospectively assessed a plasma immune-related miRNA-signature classifier (MSC) in a succeeding series of 140 patients with NSCLC before starting therapy with ICIs [46]. The MSC assessed 24 different miRNAs (miR-101-3p, miR-106a-5p, etc.) and allowed the stratification of patients into three risk levels (low, intermediate and high risk). The results of this study revealed that no patient with an MSC high risk level responded to immunotherapy; additionally, the combination of MSC and PD-L1 allowed for the identification of three risk groups of patients having 39%,18% and 0% one year PFS (*p* < 0.0001) and 88%, 44% and 0% of one year OS (*p* < 0.0001), respectively, according to the presence of 2-1-0 favorable markers. The authors concluded that plasma MSC test combined with PD-L1 tumor expression can allow for the identification of subgroups of advanced NSCLC cancer patients with different outcomes during immunotherapy.

Other Italian researchers, after a large-scale screening of 799 EV-miRNAs, recognized EV-miR-625-5p as a novel independent biomarker of response and survival in 88 patients treated with ICIs (nivolumab 35 patients and pembrolizumab 53 patients) in the first (37 patients) or second-third line settings (51 patients) [47]. The authors identified a cut-off value of miR-625-5p of 5.47 and classified 55 patients as miR-625-5p high and 33 patients as miR-625-5p low; the median PFS and OS in the miR-625-5p low and high class were, respectively, 13.2 months (95% CI 6.9–27.0) and 4.7 (95% CI 3.1–7.3) (HR 2.04, 95% CI 1.24–3.35, *p* = 0.0046) and 20.0 months (95% CI 13.0–not reached) and 8.0 months (95% CI 6.1–11.0) (HR 2.14, 95% CI 1.28–3.58, *p* = 0.0031). The authors also reported that in the multivariate analyses PD-L1 expression levels and miR-625-5p class were independently correlated with patients results. In particular, they observed that the miR-625-5p low/PD-L1 ≥ 50% group exhibited the longest median survival (27.0 months), the miR-625-5p high/PD-L1 ≥ 50% had an intermediate survival (10.75 months), while the group with PD-L1 < 50% had the briefest survival (6.3 months), independently from the miR-625-5p status. Likewise, the miR-625-5p low/PD-L1 ≥ 50% group had the most extended median survival (24.0 months), the miR−625−5p high/PD-L1 ≥ 50% had an intermediate survival (7.45 months), while the groups with PD-L1 < 50% showed the shortest survival. The authors concluded that EV-miR-625-5p associated with PD-L1 testing could let the oncologists identify in advance NSCLC patients that would benefit from ICIs. Zhang et al. evaluated the correlation between exoPD-L1 and IHC PD-L1 status and the pathological features of 29 NSCLC patients receiving anti PD-L1 treatment [48]. They showed that exoPD-L1 levels was correlated with IHC PD-L1 status and that there was a link between exoPD-L1 levels and lymph node metastasis, while IHC PD-L1 status was not associated with clinical and pathological features of the patients. Other Chinese researchers evaluated the predictive role of exoPD-L1 and CD28 in 44 patients with advanced, several types, of cancer, including 24 NSCLC, treated with ICIs [49]. The study showed that patients with high exoPD-L1 and low CD28 had short PFS indicating that the combined levels of baseline exoPD-L1 and CD28 can be useful indicators of predictive efficacy of anti-PD1 therapy.

Table 4 summarizes the previous reported experiences that evaluated TDEs/exosomal miRNAs as biomarkers in lung cancer.

Chen et al. reported that the level of PD-L1 on the EVs was notably higher in patients with metastatic melanoma than in healthy donors [50]. They also observed that the level of exoPD-L1 positively correlates with that of IFN-γ, that its basal level was remarkably higher in patients who did not respond to the anti-PD-1 treatment with ICIs and that it increased significantly, mostly within six weeks of therapy, in clinical responders. Italian researchers isolated EVs from the plasma of 71 metastatic melanoma patients and associated the amount of EVs PD-L1+ and EVs PD1+ responding to ICIs [51]. They noticed that the level of EVs PD-L1+ released from a melanoma and CD8+T cells had statistically lower levels in responders than in non-responders. French researchers evaluated exoPD-L1 levels in 46 melanoma patients during their treatment including immunotherapy and BRAF-MEK inhibitors [52]. Firstly, they proved that the level of PD-L1 was prominently higher in exosomes when compared to soluble PD-L1 or tumor biopsies. They then demonstrated that, although baseline exoPD-L1 was not correlated with clinical and pathological characteristics, their variation was associated with a tumor’s response to treatment. The authors quantified an optimal cut-off and found that the ΔexoPD-L1 cut-off >100 pg/mL had an 83% sensitivity, a 70% specificity, a 91% positive predictive value and a 54% negative predictive value for disease progression. Furthermore, the utilization of the cut-off permitted to identify two groups of patients characterized by different progression free survival (PFS) and OS. Theodoraki et al. showed that levels of exoPD-L1, but not of sPD-L1, are correlated with disease progression in neck squamous cell carcinoma (HNSCC) patients [53]. In another study, conducted by the same author in 18 HNSCC patients that were given a combination of cetuximab, ipilimumab and radiotherapy, CD3(-)CTLA4+ TDEs decreased only in disease-free patients (*p* < 0.05), while in patients with a recurrence these levels remained unaltered [54]. Therefore, the authors suggested that patients with high values of CD3(-)PD-L1+ and CD3(-)CTLA4+ TDEs at baseline may benefit from immunotherapy. Finally, American researchers reported that the decreased release of circulating CD9+/GFAP+/SVN+ and CD9+/SVN+ exosomes in patients with malignant glioma, receiving anti-survivin immunotherapy, may be correlated with longer progression free survival [55].

Our institution too is carrying out a study that aims to assess the predictive and prognostic role of TDEs in NSCLC patients treated with ICIs.

## 5. Conclusions

In the last few years, the “immunotherapy tsunami” has arrived upon us regarding therapy for NSCLC, with the prognosis of these patients finally beginning to improve. However, not all patients respond to immunotherapy and until now, even though we know that they are limited by several technical and biological issues, IHC PD-L1 expression and TMB represent the only available biomarkers in clinical practice. For all these reasons there is an urgent need to identify novel biomarkers that can help us to improve the selection of responders to immunotherapy. Liquid biopsy already has a recognized role as a useful alternative to conventional tissue biopsies for certain kinds of analysis, and in this context TDEs “the latest liquid biopsy family members”, as defined by Christian Rolfo in his editorial in the Journal of Thoracic Oncology, represent a promising source of biomarkers, such as PD-L1, mRNAs and miRNAs [56]. Obviously, blood sample collection is more convenient and less invasive when compared to tissue samples, overcoming the problems related to tumor heterogeneity and can be performed at different times during the treatment. As previously mentioned, several studies have already demonstrated both the feasibility of exo-PD-L1 measurement and its association with response to ICIs in patients with melanoma and NSCLC. Finally, other studies exhibited not only the involvement of miRNAs in several biological activities, including cell proliferation, differentiation, migration and disease progression but also their possible role as a biomarker for lung cancer patients mainly due to their stability and specificity. 

In conclusion, TDEs carry several potential biomarkers in cancer patients, including NSCLC patients, treated with immunotherapy; however, further larger studies are needed for a better definition of their role. Last but not least, in view of the promising results related to their use as a natural delivery vehicle for therapeutic agents, in the near future exosomes could also be used in anti-cancer treatment.

## Figures and Tables

**Table 1:** Possible effects of Tumor-Derived Exosomes (TDEs).

Effect	Mechanism
Improvement of cellular survival	Escape from apoptosis
	Escape from immune-surveillance
Angiogenesis	Activation of coagulation system
	Transfer of mRNA coding for growth factors
Metastasis	Transfer of oncogenes
	Matrix metalloproteinase (MMP) activity

**Table 2:** Potential applications of exosomes in NSCLC.

Diagnosis [15]
Prognosis [28,31,32]
Monitoring therapeutic efficacy [36]
Drug delivery system [35,37]
Vaccine [35,37]

**Table 3:** Main exosome isolation techniques.

Methods	Advantages	Disadvantages
Ultracentrifugation	The gold standard for protein detection	Time-consuming Exosomes may be damaged
Ultrafiltration	Short processing time and easy procedure	Small sample volume limitations Protein contamination
Chromatography	Suitable for isolation from complex biofluids	Time-consuming Small sample volume limitations
Density gradient centrifugation	High yield	Time-consuming No absolute separation of vesicles subpopulations
Immunomagnetic beads	High isolation purity	Only for exosomes with specific markers
Size-exclusion chromatography	High isolation purity	Poor selectivity compared to other chromatographic techniques
Microfluidic chip	High recovery and purity Short time-consuming	Requires sophisticated technology
Microfluidic method combined with immune separation	Simultaneous separation extraction, purification and targeted protein analysis	Only for exosomes with specific markers

**Table 4:** Tumor-derived exosomes (TDEs)/exosomal miRNAs (exomiRNAs) as biomarkers in lung cancer.

Author	Ref.	exomiRNAs	TDEs
Huang	[30]	miR-34c-3p	
Liu	[31]	miR-23b-3p, miR-10b-5p,	
		miR-21-5p	
Cazzoli	[32]	miR-378-a, miR-379, etc.	
Marconi	[33]	miR-130a-3p	
		(in association with fibrinopeptide A)	
Li	[34]	miR-184, miR-3913-5p	
Yang	[39]		PD-L1 mRNA, exoPD-L1
Li	[40]		exoPD-L1
Del Re	[41]		PD-L1 mRNA
de Miguel-Perez	[43]		exoPD-L1
Peng	[44]	has-miR-320b/-320c/-320d,	
		has-miR-125b-5p	
Halvorsen	[45]	miR-215-5p, miR-411-3p, miR-493-5p,	
		miR-494-3p, miR-495-3p, etc.	
Pantano	[47]	miR-625-5p	
Zhang Z	[48]		exoPD-L1
Zhang C	[49]		exoPD-L1
			(in association with CD28)

## Data Availability

Not applicable.
